# *In Vitro* Analyses Reveal the Effect of Synthetic Cytokinin Forchlorfenuron (FCF) on a Septin-Like Protein of Taeniid Cysticerci

**DOI:** 10.1155/2019/8578936

**Published:** 2019-03-03

**Authors:** Diana G. Rios-Valencia, Edgar O. López-Villegas, Dylan Diaz Chiguer, Adrian Marquez Navarro, Ruben D. Díaz-Martín, Benjamin Nogueda-Torres, Javier R. Ambrosio

**Affiliations:** ^1^Departamento de Parasitología, Escuela Nacional de Ciencias Biológicas-Instituto Politécnico Nacional, 11340 Ciudad de México, Mexico; ^2^Departamento de Microbiología y Parasitología, Facultad de Medicina, Universidad Nacional Autónoma de México, 04510 Ciudad de México, Mexico; ^3^Central de Instrumentación de Microscopia, Escuela Nacional de Ciencias Biológicas, Instituto Politécnico Nacional, 11340 Ciudad de México, Mexico; ^4^Clínica de Especialidades Indianilla, Dirección Médica, Instituto de Seguridad y Servicios Sociales de los Trabajadores del Estado, 06720 Ciudad de México, Mexico; ^5^Comisión Federal para la Protección contra Riesgos Sanitarios, 03810 Ciudad de México, Mexico

## Abstract

Cytokinin forchlorfenuron (FCF), a synthetic cytokinin, has been used specifically for the characterization of septins. In spite of genomic evidence of their existence, nothing is known about septin filaments in taeniid cestodes. The aim of this work was to determine the presence of a septin-like protein in cysticerci of* Taenia crassiceps* and* Taenia solium* using the deduced amino acid sequence of* T. solium* septin 4 (SEPT4_Tsm), to design and synthesize a derived immunogenic peptide (residues 88 to 103), to prepare a specific rabbit polyclonal antibody, and to examine the effects of FCF at different concentrations and exposure times on an* in vitro* culture of* T. crassiceps* cysticerci.* In vitro*, FCF altered the morphology and motility of* T. crassiceps* cysticerci, and its effects were reversible under specific concentrations. In addition, we observed by ultrastructural observation that FCF alters the cellular subunit of the protonephridial system of cestodes, where disruption of the axoneme pattern of flame cells was observed. The rabbit polyclonal antibody prepared against the synthetic peptide recognized a major band of 41 kDa in both parasites. Our results establish the importance of SEPT4_Tsm in the dynamics and survival of taeniid cysticerci, as well as their susceptibility to FCF. This is also the first report that a septin is present in the cytoskeleton of taeniids.

## 1. Introduction

Together with actin, intermediate filaments, and microtubules, septins are considered the fourth component of the cytoskeleton. They have been shown to play important biological roles in cytokinesis, recruitment of proteins, and organization/reorganization of cytoskeletal proteins. They possess a molecular mass of typically 30–65 kDa and GTPase activity domains. Based on sequence homology and the number of coiled-coil domains, mammalian septins are classified into four subgroups: septin 2 (septins 3, 9, and 12), septin 6 (septins 6, 8, 10, 11, and 14), and septin 7 (septins 7 and 13) [1, 2]. Experimentally, septins have been detected in only two helminths,* Schistosoma mansoni* [3] and* Caenorhabditis elegans* [4]. The synthetic cytokinin forchlorfenuron (N-(2-chloro-4-pyridyl)-N9-phenylurea or C_12_H_10_ClN_3_O), known as FCF, has been shown to alter the stability and function of septin filaments in yeast and mammals [5, 6]. In parasites, FCF was found to affect the function of septins only in the trematode* S. mansoni* [7].

*Taenia crassiceps* tapeworms are excellent experimental models for the study of cysticercosis and taeniasis caused by* Taenia solium* parasites [8].* T. crassiceps* cysticerci of the ORF strain can be recovered from the peritoneal cavity of experimentally infected mice, resulting in a useful laboratory model compared to larval developmental stages of other tapeworms, such as* Echinococcus granulosus*,* Hymenolepis nana*, and* T. solium*. Furthermore,* T. crassiceps *cysticerci of the ORF strain do not infect humans and are easy to study [9, 10]. This characteristic has facilitated the search for molecules with potential pharmacological, diagnostic, or immunotherapeutic value against parasitic diseases [11, 12]. Owing to its morphological and cellular similarity to* T. solium, T. crassiceps* has enabled the successful characterization of cytoskeletal proteins and changes to their expression pattern in response to antihelminthic drugs [13, 14] or other potential antiparasitic chemicals [15].

The morphology of* T. crassiceps* ORF strain cysticerci is simple: cysts are dynamic vesicles filled with clear vesicular fluid, limited by a continuous tissue. Under the microscope, they appear to be constituted of a syncytial tegumental layer that is crucial for the exchange of nutrients and waste, as well as maintenance of the host-parasite relationship [16]. As their size allows for easy microscopic examination, cysticerci can be readily checked for any alteration to their continuous movements. Thus, changes to the classical morphology of cestodes could derive from alterations to the appearance of subtegumental and flame cells, as well as the complex and intricate protonephridial ducts visible by electron microscopy. As with actin, tubulin, myosin, and paramyosin [17–20], the effect of any compound on the expression of cytoskeletal proteins can be assessed also in the cysticerci of the* T. crassiceps *ORF strain.

In tapeworms, the biological importance or the expression of septin proteins remains unknown. This is in contrast with the reported existence of septin genes in the genomes of cestodes and hence a possible role of their products in these organisms. This conundrum is exacerbated by the fact that some of the septin genes seem to possess canonical regions, while others appear incomplete. Assuming that these genes are indeed expressed, the resulting proteins are likely to be susceptible to FCF. Therefore, the aim of the present work was to perform* in vitro* characterization of a possible septin in the larval stage of the* T. crassiceps* ORF strain and in* T. solium.* This study yields a preliminary description of taeniid septins and establishes their importance in the biology of these tapeworms, particularly in view of possible targeted drugs.

## 2. Materials and Methods 

### 2.1. Bioinformatics Analysis of* T. solium* Septin 4

The SEPT4_Tsm sequence of* T. solium* was obtained from GeneDB (http://www.genedb.org) under accession number TsM_000487200. A protein BLAST search of the sequence was carried out in UniProt using the NCBI database. Using the Clustal Omega Multiple Sequence Alignment database, sequences of SEPT4_Tsm, SEPT7_Tsm, SEPT_Tsm, and SEPT10_Tsm were aligned and compared with homologous sequences from* S. mansoni* and human. We then identified the septin motifs [G1 (GXXXXGKS/T), G3 (DXXG), and G4 (XKXD)], SUE domain, and coiled-coil structure of each sequence.

### 2.2. Design and Production of SEPT4_Tsm Synthetic Peptide and Preparation of Polyclonal Antibody

Using the amino acid sequence of* T. solium* SEPT4_Tsm, we designed an immunogenic peptide using the Emini Surface and Kolaskar and Tongaonkar algorithms at the IEDB site (http://www.iedb.org). To predict the accessibility and immunogenicity of the peptide present in the SEPT4_Tsm sequence, the peptide EPYYAEYANVGGVGEK, located between positions 88 and 103 of SEPT4_Tsm, was synthesized by GenScript Biology Co. (Piscataway, NJ, USA) with desalted purity and a C-terminal modification of four MAPS. Polyclonal anti-taeniid antibodies were produced by scheduled immunizations of New Zealand rabbits with 250 *μ*g/mL of the peptide resuspended in complete Freund adjuvant, followed by three additional immunizations with the same amount of antigen dissolved in incomplete Freund adjuvant and phosphate-buffered saline (PBS) applied at 8-day intervals. Antibody recognition was assessed after each immunization at ratios of 1:50–1:10,000 against parasite protein extract.

### 2.3. Parasites

The larval stage parasitic forms of taeniids were used for the present studies. Cysticerci of* T. crassiceps* ORF strain were recovered after three months of infection, from the peritoneum of experimentally infected BALB/c female mice of 5–7 weeks of age, whereas* T. solium* cysticerci were dissected from naturally infected pigs. After their recovery, the parasites were washed with PBS (pH 7.2), homogenized (PRO200; Pro Scientific Inc., Oxford, CT, USA) in the presence of ice, and resuspended in cytoskeletal buffer (6.7 mM phosphate, 0.04 M KCl, 1 mM MgCl_2_, pH 7.4) [21] complemented with a commercial cocktail of protease inhibitors (Complete Protease Inhibitor Cocktail Tablets; Roche, Mannheim, Germany). Later, parasites were frozen at -70°C until required. The study was approved by the Ethics and Research Committees of the Facultad de Medicina, Universidad Nacional Autónoma de México (#IN216213).

### 2.4. Effect of FCF on Whole Cysticerci

After recovering the cysticerci from the peritoneal cavity of mice, they were washed exhaustively with sterile PBS (1 M, pH 7.4), prior to a final wash with a mixture of antibiotic-antimycotic reagents (100×; GIBCO®, Invitrogen, Carlsbad, CA, USA) in PBS. Then, ten parasites per well were added to a 24-well plate. Each well contained 2 mL of RPMI 1640 medium (Merck-Millipore, Bedford, MA, USA) supplemented with 3.4 mM HEPES and 11.9 mM of sodium bicarbonate and antibiotic-antimycotic solution. To evaluate the effect of FCF (sc-204759A; Santa Cruz Biotechnology, Dallas, TX, USA), 500, 50, and 5 *μ*M working dilutions were prepared from a stock solution of FCF (500 mM dissolved in DMSO). Control groups of parasites were maintained in RPMI 1640 medium supplemented with only 0.1% DMSO. Cysticerci were incubated for different periods (0.25, 0.5, 1, 5, and 24 h) at 37°C and a relative humidity of 95%. Observations were performed on an Olympus SZx7 microscope (Olympus, Tokyo, Japan) at a 7:1 zoom ratio, coupled to a CANON EOS Rebel T31 camera (Canon, Tokyo, Japan). Videos were edited using FinalCutPro X software. Images and videos were taken at different times during cultivation. The metabolic activity of cysticerci was assessed by biotransformation of Alamar blue® reagent (Invitrogen). Spectrophotometric readings of the recovered supernatants were recorded at 500 nm on an ELx808™ Absorbance Microplate Reader (BioTek, Winooski, VT, USA), and densitometry values were processed and analyzed using Kruskal-Wallis and Dunnett methods. All samples were evaluated in triplicate. The reversible effect of FCF was evaluated after removing FCF by changing the culture medium to FCF-free medium, followed by observation of the motility of the cysticerci. For establishing the visual and quantitative effects of FCF on the viability of the cysticerci, we used 5 *μ*M of a commercial fluorescent dead cell stain, SYTOX Green Nucleic Acid Stain (S7020; Thermo-Fisher Scientific, Waltham, MA, USA), according to the recommendations of the manufacturer. Under this strategy, measurements of relative fluorescence units (RFUs) were read using the Synergy™ HTX Multi-Mode Microplate Reader (BioTek) with two optic filters: an excitation filter of 485/20 nm and emission filter of 528/20 nm. For use as dead control cells, cysticerci were heated to 65°C. Then, cysticerci were fixed with 4% paraformaldehyde and observed under a microscope at 20× magnification (Diaphot; Nikon, Chiyoda-ku, Tokyo, Japan) equipped with an epifluorescence accessory and the appropriate filters. The indirect effects of FCF over actin and *α*-tubulin of* T. crassiceps *cysticerci were evaluated in cryosections with phalloidin–rhodamine and commercial DM1A antibody as described in other studies performed by us [20, 22].

### 2.5. Observation of the Effect of FCF by Transmission Electron Microscopy

After 1 h of incubation,* T. crassiceps* cysticerci were recovered from control RPMI 1640 medium and medium containing two different FCF concentrations (50 and 500 *μ*M), washed with PBS, fixed with 2.5% glutaraldehyde, washed three times with PBS for 5 min, and postfixed with OsO_4_ for 1 h at room temperature. Then, the parasites were dehydrated with increasing concentrations of ethanol, preembedded in propylene-resin oxide (2:1), and treated with propylene-resin (1:1) and again with propylene-resin oxide (1:3). The entire process was performed at room temperature during 1 h. After that, cysts were embedded in epoxy resin at 60°C for a period of 24 h. Fine 70 nm sections of the parasites were obtained with an ultramicrotome and then mounted on 200 mesh copper grids. To optimize the contrast of the sectioned and embedded parasites on the copper grids, they were treated with 4% uranyl acetate and Reynolds lead citrate (Electron Microscopy Science, Mexico City, Mexico). Observations were performed on a JEOL JEM 1010 (Jeol Ltd., Tokyo, Japan) transmission electron microscope at 60 kV. Digital images were acquired using Advanced Microscopy Techniques Corp Image Capture Engine 7.0 (Woburn, MA, USA) and then processed with ImageJ 1.46r (NIH, Bethesda, MD, USA).

### 2.6. Protein Extracts

Cysticerci were homogenized in the presence of cytoskeletal buffer and sonicated three times at 50 U of amplitude, for 30 s each time and 1 min intervals, using a bath of ice (Vibra-cell 75185; Sonics and Materials Inc., Newtown, CT, USA). Then, the resultant suspension was desalted and precipitated at -20°C in a solution containing acetone, 10% tricarboxylic acid, and 20 mM dithiothreitol. The suspension was centrifuged at 28,000 ×* g* for 15 min at 4°C and the resultant pellet was recovered and resuspended in cytoskeletal buffer at 4°C as previously reported [20]. Protein concentration was determined with the DC Protein Assay reagent (Bio-Rad, Hercules, CA, USA).

### 2.7. Electrophoretic Analysis

The protein profile of the parasite extracts was obtained by sodium dodecyl sulfate polyacrylamide gel electrophoresis (SDS-PAGE) under reducing conditions (2.5% 2-mercaptoethanol) on 10% polyacrylamide gels. Standard Precision Plus Protein molecular weight markers (#161-0373; Bio-Rad) were used for reference. Proteins were separated using the PowerPac 3000 power supply (Bio-Rad) at 80 V for 30 min (stacking gel) and 120 V for 1.5 h (separating gel). The gels were then stained with Coomassie blue (PhastGel ™ Blue; GE Healthcare, Little Chalfont, Buckinghamshire, UK) and destained in the presence of acetic acid/methanol. Protein bands were analyzed using Quantity One 4.6 software (Bio-Rad).

### 2.8. Detection of SEPT4_Tsm by Immunorecognition

After electrophoretic separation of the proteins, the gels were electrotransferred to polyvinylidene fluoride membranes (Millipore) using a Trans-Blot SD Semi-Dry transfer Cell (Bio-Rad) at 15 V for 35 min. Prior to transfer, the membranes were processed as indicated by the manufacturer. After transferring the proteins, the membranes were blocked for 1 h at room temperature in the presence of 2% bovine serum albumin in PBS mixed with 0.15% Tween20. Polyclonal antibodies against SEPT4_Tsm (1:5,000) were reacted against total protein extracts of the* T. crassiceps* ORF strain and of* T. solium* cysticerci. Commercial secondary goat anti-rabbit IgG (H + L) antibody conjugated to horseradish peroxidase (HRP) (1:2,000, 81-6520; Zymed, South San Francisco, CA, USA) and goat anti-mouse IgG (H + L) antibody conjugated to HRP (1:1,000, 81-6520; Zymed) were used to detect binding. Antigen-antibody interactions were revealed by adding a chemiluminescence developmental kit solution (SuperSignal West Pico Chemiluminescent Substrate, Thermo-Fisher Scientific). Membranes were imaged using a ChemiDoc XRS system (Bio-Rad).

## 3. Results 

### 3.1. Bioinformatics Analysis of* T. solium* Septins

The search for septin genes in the genome of* T. solium* yielded three complete sequences (TsM_000487200, TsM_000458600, and TsM_000256200). These septins appeared to contain conserved septin motifs, including the GTPase motifs (G1, G3, and G4) and the SUE region. Following bioinformatics analysis of these sequences, the deduced proteins were classified into the SEPT2, SEPT6, and SEPT7 groups based on their sequence similarity to other septins from these groups (Supplementary Figure 1). The search for septins also identified two short sequences (TsM_000748600 and TsM_000242900) that, when combined, produce a complete septin sequence included in the SEPT7 group (Table 1). Complete sequences of all* T. solium *septins are shown in Supplementary Figure 1.

### 3.2. Effect of FCF in* Taenia crassiceps* ORF Strain Cysticerci

**Figure 1:**
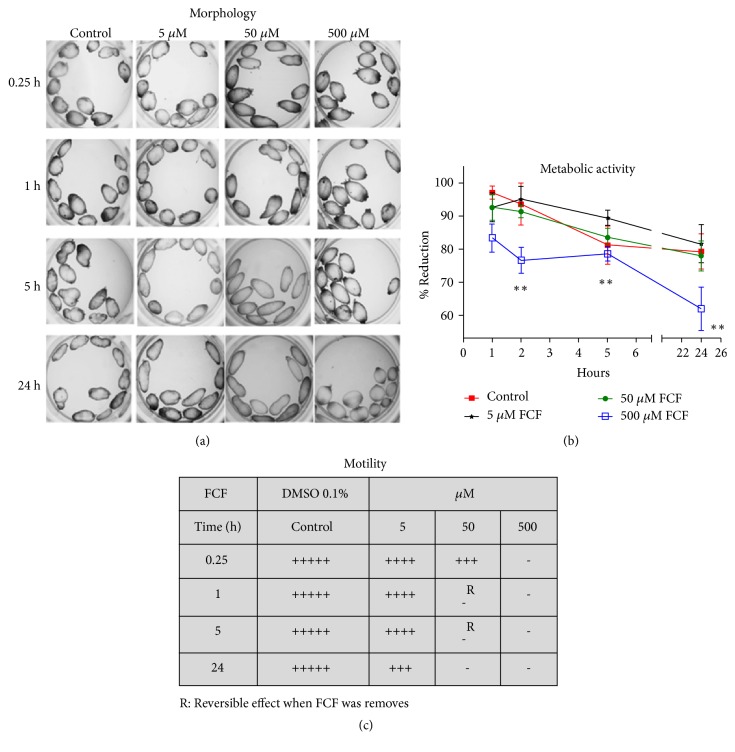
*In vitro evaluation of the effect of FCF on T. crassiceps cysticerci.* (a) Changes in the morphology of cysticerci were detected by stereomicroscopy, using three concentrations of FCF (5, 50, and 500 *μ*M) at four different times (0.25, 1, 5, and 24 h). (b) Changes in metabolic activity were measured by the reduction of Alamar blue® (Y-axis) over a period of 24 h (X-axis). (c) Table summarizing the effect of FCF on parasite motility, where R represents the reversibility of changes in motility under these conditions.

Figure 1 shows the effect of FCF treatment on the morphology (Figure 1(a)), viability (Figure 1(b)), and motility (Figure 1(c)) of cysticerci of* T. crassiceps*. Control groups consisting of untreated parasites are shown for each condition. Morphological observations revealed that untreated parasites preserved their rough surface (Figure 1(a), first column). After 1 h in the presence of 5 *μ*M FCF, the morphology and motility of parasites were akin to those of the control group (Figure 1(a), second column; Supplementary Movie 2; Figure 1(c)). In contrast, at 50 *μ*M FCF and depending on the duration of treatment, the parasites became more elongated and their surface was smooth (Figure 1(a), third column). Importantly, at this dose, motility was reduced after 15 min of culture and was completely absent at all other times (Supplementary Figure S4; Figure 1(c)). In addition, at this concentration, the effects of FCF on motility were reversible after 1 h and 5 h of treatment (Supplementary S4); however, after 24 h, motility could not be recovered (Supplementary S5). At 500 *μ*M FCF, the morphology of cysticerci was completely altered; cysticerci became oval with a very smooth surface, and multiple small invaginations appeared at the poles (Figure 1(a), fourth column). Notably, fewer such invaginations were found under other conditions. Moreover, motility was completely absent (Supplementary Figure S3; Figure 1(c)). After 24 h of treatment with 500 *μ*M FCF, the parasites looked similar to balloons (Supplementary Figure S3).

The metabolic biotransformation of Alamar blue® is shown in Figure 1(b). All groups appeared to possess a similar metabolic activity, which diminished over time; however, cysticerci treated with 500 *μ*M FCF displayed consistently lower values at all tested times, as confirmed by statistical analysis (Kruskal-Wallis and Dunnet tests).

**Figure 2:**
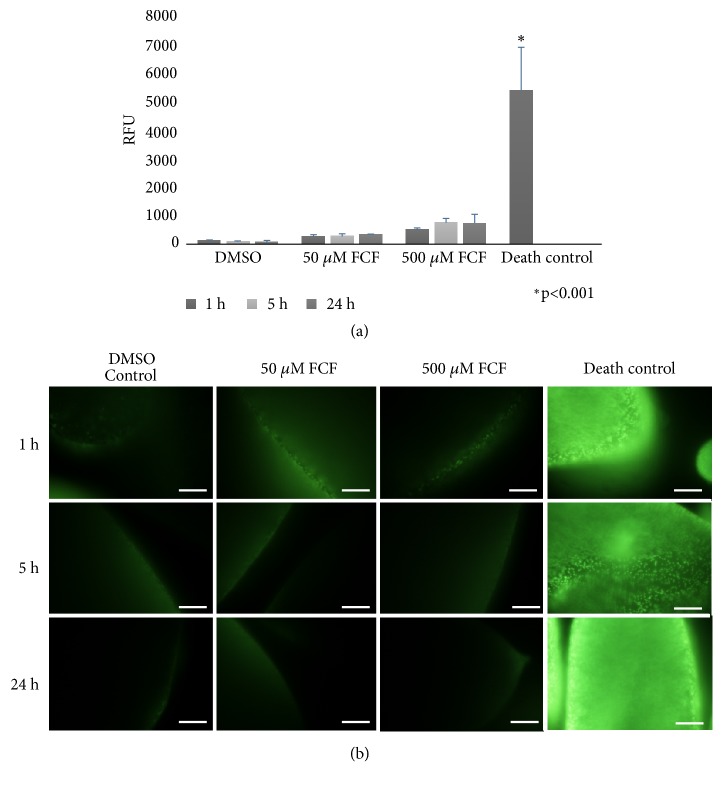
*Viability assay of cysticerci treated with 50 and 500 μM FCF*. Viability was analyzed by SYTOX green. (a) Quantification of RFUs of cells treated with DMSO control or FCF (50 or 500 *μ*M) compared with that of the dead control cells. (b) Microscopic analysis of parasites incubated with SYTOX green after FCF exposure. Scale bars represent 500 *μ*m. Bright-field images are shown in Supplementary Figure S4.

Viability was assessed by SYTOX green in live (Figure 2(a)) and fixed (Figure 2(b)) cysticerci treated with 50 and 500 *μ*M FCF. Live cysticerci exhibited significantly lower values (measured as RFU) than heated cysticerci used as dead control cells (p=0.01). After fixation of the parasites, those treated with FCF also displayed low intensities of fluorescence in comparison with those killed by heat (Figure 2(b)); parasites treated with DMSO as a control showed similar low intensities of fluorescence. Observations of the cysticerci under bright-field microscopy (Supplementary Figure S6) showed that parasites did not experience changes in size or form.

**Figure 3:**
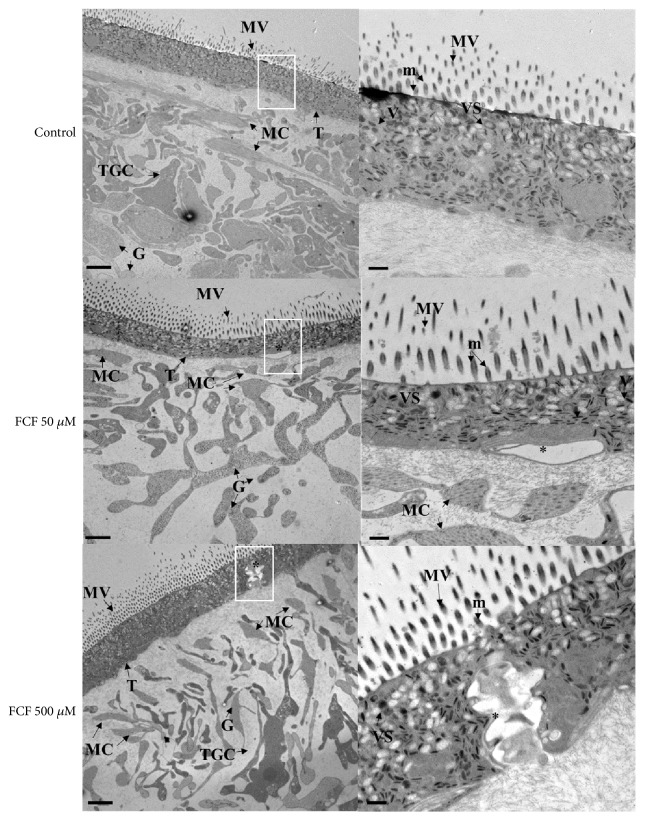
*Morphological changes caused by FCF on T. crassiceps tegument visualized by transmission electron microscopy.* MC, muscular cell; TGC, subtegumentary cell; G, glycogen; m, microtrics; MV, microvilli; V, vacuoles; VS, vesicles; T, tegument. Regions presenting changes or damage are indicated by an asterisk. Scale bar, 500 nm. The insert marked within a white box is shown at higher magnification to the left; it shows the outermost region of the tegument (scale bar, 2 nm). All cysticerci were exposed for 1 h to either 0.1% DMSO (control) or varying concentrations of FCF. The ultrastructure of the tegument in the control reveals the classic arrangement of cells and intact microvilli. In FCF-treated cysticerci, microvilli, vesicles, and microtrics are more evident.

**Figure 4:**
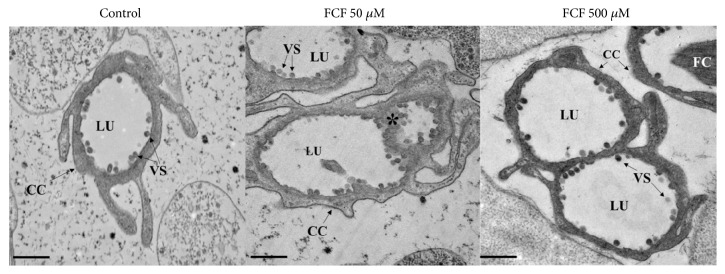
*Effect of FCF on the morphology of protonephridial ducts visualized by transmission electron microscopy.* CC, cellular channel; LU, lumen; M, mitochondria; VS, vesicles. Regions presenting structural changes are indicated by an asterisk. Scale bar, 500 nm.

**Figure 5:**
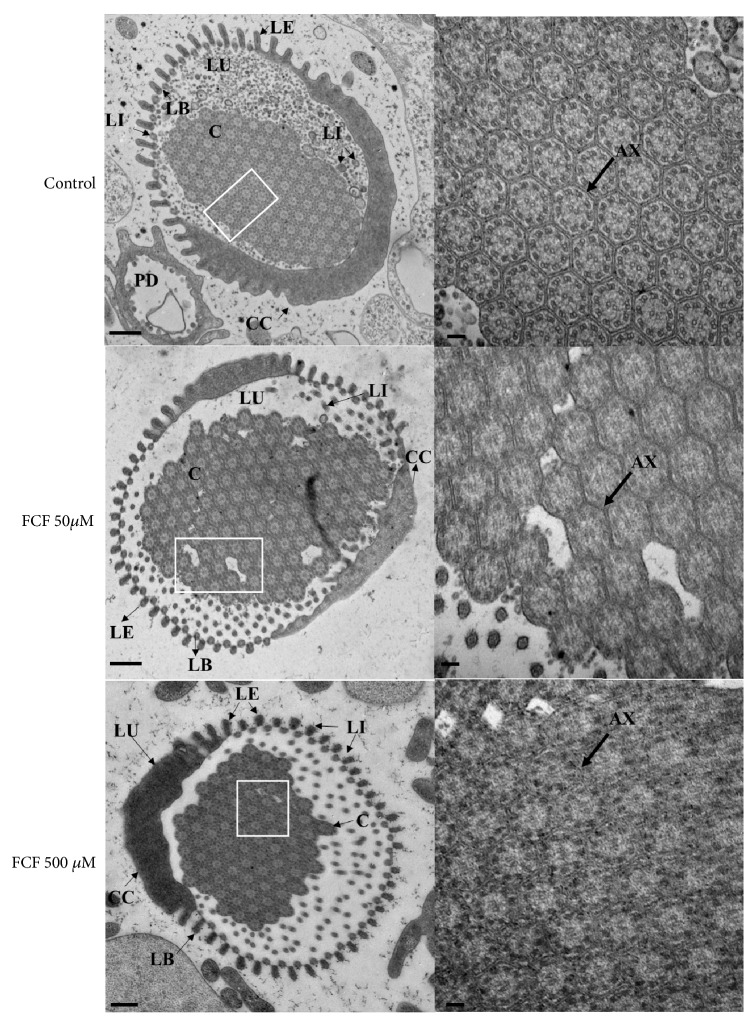
*Effect of FCF on the morphology of flame cells visualized by transmission electron microscopy.* AX, axoneme; C, cilia; CC, cell channel; LB, basal lamina; LE, leptotrich (external); LI, leptotrich (internal); LU, lumen; PD, protonephridial duct. Scale bar, 2 nm (left), 100 nm (right). Images on the left side show flame cells, with cilia and leptotrich, surrounded by cell channels; images on the right side show the distribution of cilia and the position of the axoneme (indicated by an arrow).

*Ultrastructural Analysis of the Effect of FCF on Cysticerci Tissues.* The ultrastructure of the surface and inner tissues of cysticerci was observed at different magnifications using transmission electron microscopy, as shown in Figures 3, 4, and 5. The largest changes to the morphology of the parasites were observed with the highest concentration of FCF (500 *μ*M).

Ultrastructural analysis of the exterior and interior of the tegument is shown in Figure 3. In the control cyst (upper row), the classical morphologies of the tegument and germinal layer can be seen. Treatment with 50 *μ*M FCF (middle row) and 500 *μ*M FCF (lower row) produced changes to the thickness of the tegument, increased vesiculation and the number of mitochondria, and altered cell morphology and intercellular connections in the germinal layer. In addition, changes to the morphology of muscular and glycogen cells, but not to the morphology of microvilli, were observed. Treatment with 500 *μ*M of FCF caused disruption of the tegument, with very large invaginations protruding from the basal membrane towards the surface. These invaginations either ripped through the tegument or were observed along its length. Further below (Figure 4), treatment with 500 *μ*M FCF made the protonephridial ducts appear more slender than those of control animals. In spite of their appearance, the ducts were more electrodense than those of controls. Finally, treatment with FCF (50 *μ*M, middle row and 500 *μ*M, bottom row in Figure 5) produced important morphological alterations of flame cells and the components of the ciliary tuft (left column in Figure 5). While in the control cyst (upper row in Figure 5), the cilia in the ciliary tuft presented a 9 + 2 conformation (seen at higher magnification in the right column of Figure 5), FCF caused the cilia to become more compacted and disordered. Thus, treatment with 500 *μ*M FCF resulted in the loss of the 9 + 2 conformation (lower row to the right in Figure 5).

### 3.3. Taeniid Septin Immunorecognition

**Figure 6:**
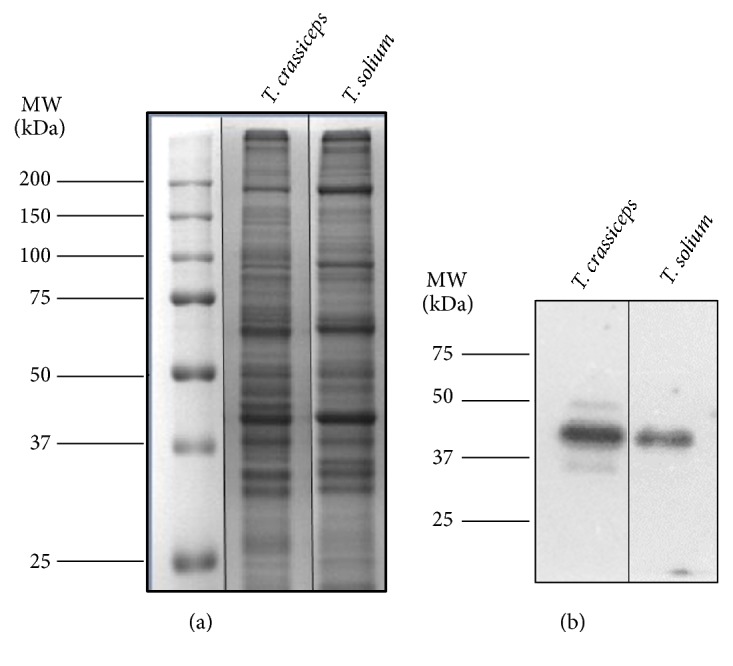
*Electrophoretic and immunochemical analysis of taeniid septin-like proteins.* (a) SDS-PAGE of the* T. crassiceps* and* T. solium* protein extracts stained with Coomassie blue. Molecular weight (MW) markers are indicated in the first lane. (b) Immunorecognition with the anti-SEPT4_Tsm antibody in* T. crassiceps* and* T. solium* protein extracts. Evaluation was performed by chemiluminescence following western blotting.

After resolving the proteins in cytoskeletal extracts using SDS-PAGE under reducing conditions (Figure 6(a)), they were transferred to membranes and single bands for each taeniid were recognized by anti-SEPT4_Tsm (Figure 6(b)) antibody. The anti-SEPT4_Tsm antibody detected a band with an apparent molecular weight (MW) of 41 kDa in each of the two taeniid species, suggesting it may correspond to SEPT4_Tsm.

## 4. Discussion

As mentioned earlier, the participation of septins in the cellular biology of parasites is important for the adaptation and survival of these organisms inside their hosts; therefore, it is the key to understand their expression. In the trematode* S. mansoni*, use of recombinant proteins and confocal microscopy have revealed the existence of four septins [3]. However, no such information has been available for cestodes. In the present study,* in silico* analysis of the* T. solium* genome revealed the existence of genes that appeared to encode septins. Classic septin domains were deduced from putative septin sequences and bioinformatics analysis indicated that they were homologous to septins from other species, including humans (Supplementary Figure S1). A comparison of the deduced protein sequences of* T. solium* septins with those of* S. mansoni* and human suggested that the taeniid septins belonged to three major septin groups: 2, 6, and 7.

FCF acts by inhibiting the polymerization of septin filaments; it has been proposed to bind to the G domains, which are important sites for the hydrolysis of GTP to GDP and also for septin-septin interactions [23]. When bound to FCF, septin filaments lose functionality, thus blocking normal cell migration and cytokinesis [6]. In addition, it has been demonstrated that there is an intrinsic relationship between the actin cytoskeleton and the rearrangement of septin 2 in NRK cells, meaning that any change induced in one of these cytoskeletal proteins is sufficient for altering the stability and functionality of the other [24].

In this study, we focused on the effect of FCF on cysticerci of the metazoan* T. crassiceps* at a morphological and functional level. We hypothesized that the compound could disrupt the interaction between septins due to FCF competing with GTP, leading to inadequate formation of filament septins [25]. The reduction in the parasites' dynamics and motility at increasing FCF concentrations (Supplementary Figures S2 and S3) could be an effect of that interaction. Eventually, after prolonged* in vitro* treatment at the highest FCF concentration, the parasites were reported dead. This effect of FCF on taeniid parasites is interesting, as until now, septins were not reported in these organisms.

Confocal microscopy observations in the trematode* S. mansoni *[3] revealed the presence of septins on the surface and in the most adjacent muscle layers of the tegument. There, septins were proposed to interact with muscle fibers expressing actin filaments. Additionally, in the same study, septins were detected at osmoregulatory sites of the protonephridial ducts. In metazoans [26], septins are thought to be important for the formation and maintenance of cilia, meaning that they are required for the regulation of ciliogenesis and ciliary transport via microtubule dynamics and vesicle transport. The interaction of septins with other cytoskeletal proteins was reported in mammalian cells [27, 28]. In the present study, the effect of FCF on the morphology (Figure 1) and reduced motility in cysticerci of* T. crassiceps* (Supplementary Figures S2 and S3) indicated that septins may interact with the actin and myosin located in these tissues. Actin and muscular myosin are highly expressed in the tissues of cestodes, as they are required for the motility and muscular contraction characteristic of these organisms [17, 29]. Therefore, if those cytoskeletal elements interact with septins, any alterations to the latter are likely to affect the organisms' motility, as already proposed for* S. mansoni* [7]. In the present study, changes in the fluorescence of polymerized actin or tubulin were observed in cysticerci tissues following FCF treatment (Supplementary Figure 7). These gross observations may indicate that FCF-induced alterations in SEPT4_Tsm disrupt the polymerization of actin and tubulin in* T. crassiceps *cysticerci, as we have previously shown [20]. Future studies on the interactions between septins and these cytoskeletal proteins should demonstrate the exact nature of this relationship and its importance for the survival of cysticerci.

The cestode tegument is crucial for the survival of these parasites as it allows for transport, exchange of molecules, and elimination of waste [16]. Therefore, any alteration that affects its stability and dynamics is potentially lethal for these metazoan organisms, as observed already in cysticerci and adult tapeworms of* Taenia* species treated with antihelminthic drugs [14, 30, 31]. Here, incubation with FCF at concentrations of 50 and 500 *μ*M for 1 h produced visible changes along the tegument, in the form of either morphological alterations or clear disruptions that compromised the integrity of the syncytial tegument (Figure 4). We speculate that, at this point, the parasites might have become damaged and have lost the capacity to maintain a regulated exchange of substances. This, in turn, would have led to the observed complete loss in motility (Supplementary Figures S2 and S3) and metabolic functionality (Figure 1(b)). If* Taenia* septins are the target of FCF and these proteins are involved in maintaining the homeostasis and vitality of the parasites, then the effect of FCF could arise from alterations to septins and the ensuing repercussions on the parasites.

In addition to the effect of FCF on the tegumental regions, other cysticerci tissue regions, such as the protonephridial system, were also affected by FCF. Indeed, FCF affected the protonephridial ducts and flame cells, both of which are important because they are involved in essential osmoregulatory functions. These functions allow the parasites to maintain internal water and salt equilibrium through the circulation of fluids inside protonephridial ducts, pushed by the normal ciliary movements of flame cells. Based on electron microscopy observations, the protonephridial ducts (Figure 4) became fused and lost their capability to maintain fluid circulation. These changes were accompanied by morphological alterations to flame cells (Figure 5), akin to those reported following treatment with antihelminthic drugs [13]. Together, these changes could dramatically alter the dynamics of fluid movements, resulting in intoxication and death of the parasites. This hypothesis is supported by the observed alteration of the cyst's overall morphology (Figure 1(a)), reduced viability (Figure 2), and loss of motility (Supplementary Figures S2 and S3). Interestingly, FCF affected the composition of cilia inside the ciliary tufts (Figure 5), with loss of the canonical 9 + 2 distribution of cilia. This finding suggests that these cells were no longer able to perform normal ciliary movements and expel the fluids from protonephridial ducts. Accordingly, cysticerci were likely unable to maintain the osmotic pressure of their tissues, a condition that affected the parasites' viability, as after treatment with androgens [22]. As established for* S. mansoni* [3], septins are highly expressed in protonephridial ducts and flame cells; therefore, any alteration to the structural morphology of these regions in cysticerci following FCF treatment would be indicative of the sites of action of FCF.

In the present study, a search for taeniid septins yielded the sequence of a gene corresponding to septin 4; this septin belongs to group SEPT2 in humans [32]. Comparison of the amino acid residues of the deduced septin (SEPT4_Tsm) with those of human septin 2 (notably, there is no published genome of* T. crassiceps*) showed an average identity of 57%. BLAST analysis of the sequence of human septin 2 against the entire genome of* T. solium* revealed that the protein with the highest percent identity corresponded to SEPT4_Tsm (data not shown).

The anti-SEPT4_Tsm antibody recognized specifically a single band of the same MW (41 kDa) in both taeniids (Figure 6(b)). Given that the MW deduced from the genome of* T. solium* is 65 kDa, this discrepancy could be explained by the existence of other septin genes in the genome of* T. solium*, expression of an unknown septin protein or recognition of an isoform of SEPT4_Tsm. It is also important to mention that the search for the most immunogenic peptide to use in antibody production was geared towards the highest specificity in the recognition of* Taenia* septins. Future experiments should thus consider the use of other methodologies such as mass spectrometry to confirm the identity of this protein.

## 5. Conclusions

The effects of FCF are useful for establishing the presence of septins in taeniid cestodes. FCF treatment altered the morphology, viability, and motility of* T. crassiceps* cysticerci, owing to its action on septin-like proteins. The molecular identity of those septin-like proteins was confirmed by the use of polyclonal antibody designed specifically against taeniid septins. As septins are important for the cytoskeletal organization of taeniids, the action of FCF indicates that septins fulfill vital functions in tapeworms. Accordingly, FCF or its derivatives could serve as potential antihelminthic drugs.

## Figures and Tables

**Table 1:** Sequences of septins in *Taenia solium.*

Accession number	Septin	Septin group
TsM_000487200	Septin 4	SEPT2

TsM_000458600	Septin 7	SEPT7

TsM_000256200	Septin 10	SEPT6

TsM_000748600^1^	Septin	SEPT7

TsM_000242900^1^	Septin	SEPT7

^1^The joining of these sequences produces a complete septin sequence. The resulting septin, based on its similarity to other septin sequences, was considered to belong to the SEPT7 group.

## Data Availability

All data generated or analyzed during this study are included in this published article. The datasets used and analyzed during the current study are available from the corresponding author on reasonable request.

## Supplementary Materials

Supplementary Materials Supplementary Figure S1.* Alignment of T. solium septins*. A: SEPT4_TsM, B: SEPT7_Tsm, C: SEPT_Tsm, and D: SEPT10_Tsm were aligned with* S. mansoni* septins and human septins. The conserved septin motifs (G1, G3, G4, and SUE) are enclosed by black boxes. The percent similarities are shown at the bottom of the figure. Supplementary Figure S2.* Motility of cysticerci treated with 5, 50, and 500 μM FCF compared to control after 1 h of treatment*. Motility in cysticerci was reduced upon exposure to the highest concentrations of FCF (50 and 500 *μ*M) in comparison with that of the control parasites (treated with DMSO), which exhibited normal motility. Supplementary Figure S3.* Motility of cysticerci treated with 5, 50, and 500 μM FCF compared to control after 24 h of treatment.* Upon exposure to the highest concentrations of FCF (50 and 500 *μ*M), motility was reduced in comparison with that of the control parasites (treated with DMSO), which exhibited normal motility. Supplementary Figure S4.* Reversible effect of treatment with 50 μM FCF compared to control after 5 h of treatment.* Cysticerci recovered their motility slowly after changing the medium to FCF-free medium. Supplementary Figure S5.* Reversible effect treatment with 50 μM FCF compared to control after 24 h of treatment.* After 24 h of exposure to FCF, cysticerci did not recover their motility after changing the medium to FCF-free medium. Supplementary Figure S6.* Viability of cysticerci treated with 50 and 500 μM FCF.* Bright-field observations. Viability was analyzed by SYTOX green. (A) Quantification of RFUs of cysticerci treated with DMSO (control) or FCF (50 or 500 *μ*M) compared with that of dead control cells. (B) Microscopic analysis of parasites incubated with SYTOX green after FCF exposure. Scale bars represent 500 *µ*m. Supplementary Figure S7.* Indirect effect of FCF on actin and tubulin in cysticerci of T. crassiceps.* Cysticerci were treated with 500 *μ*M FCF and then evaluated for the expression of actin using phalloidin (red) and of tubulin using anti-DM1A antibody (green). We observed changes in the expression of these proteins in comparison with that in the DMSO-treated control group. Arrows indicate the tegument (T) and VS (vesicular fluid). Scale bar represents 500 *µ*m.
