# Supplementary figures and images for: In Vitro Analyses Reveal the Effect of Synthetic Cytokinin Forchlorfenuron (FCF) on a Septin-Like Protein of Taeniid Cysticerci
Source: J Parasitol Res. 2019 Mar 3;2019:8578936. doi: 10.1155/2019/8578936 (PMC6420996; doi:10.1155/2019/8578936)

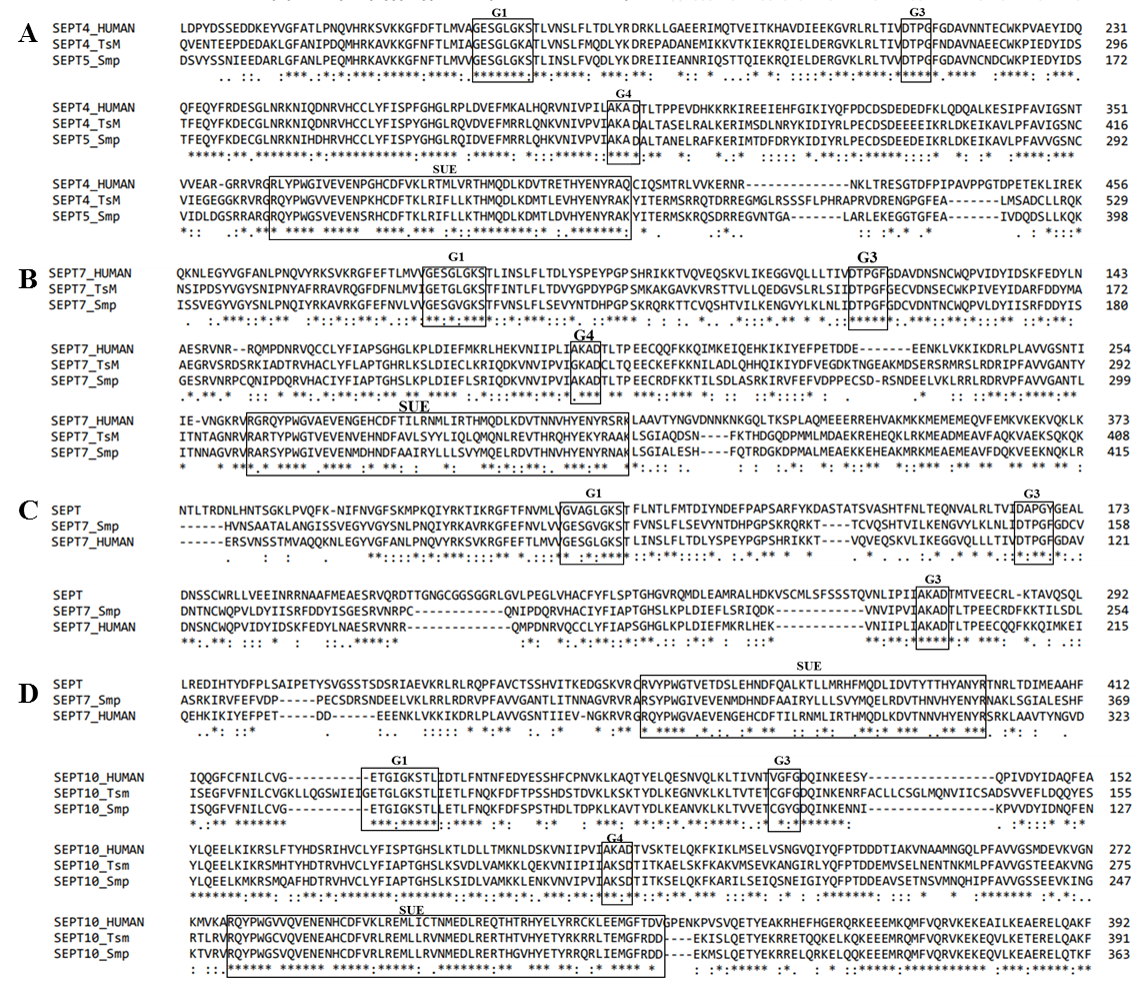

Supplement: Supplementary Materials — Supplementary Figure S1. Alignment of T. solium septins. A: SEPT4_TsM, B: SEPT7_Tsm, C: SEPT_Tsm, and D: SEPT10_Tsm were aligned with S. mansoni septins and human septins. The conserved septin motifs (G1, G3, G4, and SUE) are enclosed by black boxes. The percent similarities are shown at the bottom of the figure. Supplementary Figure S2. Motility of cysticerci treated with 5, 50, and 500 μM FCF compared to control after 1 h of treatment. Motility in cysticerci was reduced upon exposure to the highest concentrations of FCF (50 and 500 μM) in comparison with that of the control parasites (treated with DMSO), which exhibited normal motility. Supplementary Figure S3. Motility of cysticerci treated with 5, 50, and 500 μM FCF compared to control after 24 h of treatment. Upon exposure to the highest concentrations of FCF (50 and 500 μM), motility was reduced in comparison with that of the control parasites (treated with DMSO), which exhibited normal motility. Supplementary Figure S4. Reversible effect of treatment with 50 μM FCF compared to control after 5 h of treatment. Cysticerci recovered their motility slowly after changing the medium to FCF-free medium. Supplementary Figure S5. Reversible effect treatment with 50 μM FCF compared to control after 24 h of treatment. After 24 h of exposure to FCF, cysticerci did not recover their motility after changing the medium to FCF-free medium. Supplementary Figure S6. Viability of cysticerci treated with 50 and 500 μM FCF. Bright-field observations. Viability was analyzed by SYTOX green. (A) Quantification of RFUs of cysticerci treated with DMSO (control) or FCF (50 or 500 μM) compared with that of dead control cells. (B) Microscopic analysis of parasites incubated with SYTOX green after FCF exposure. Scale bars represent 500 µm. Supplementary Figure S7. Indirect effect of FCF on actin and tubulin in cysticerci of T. crassiceps. Cysticerci were treated with 500 μM FCF and then evaluated for the expression of actin using phalloidi [file 8578936.f1.zip › 8578936.f1/S1.tif]

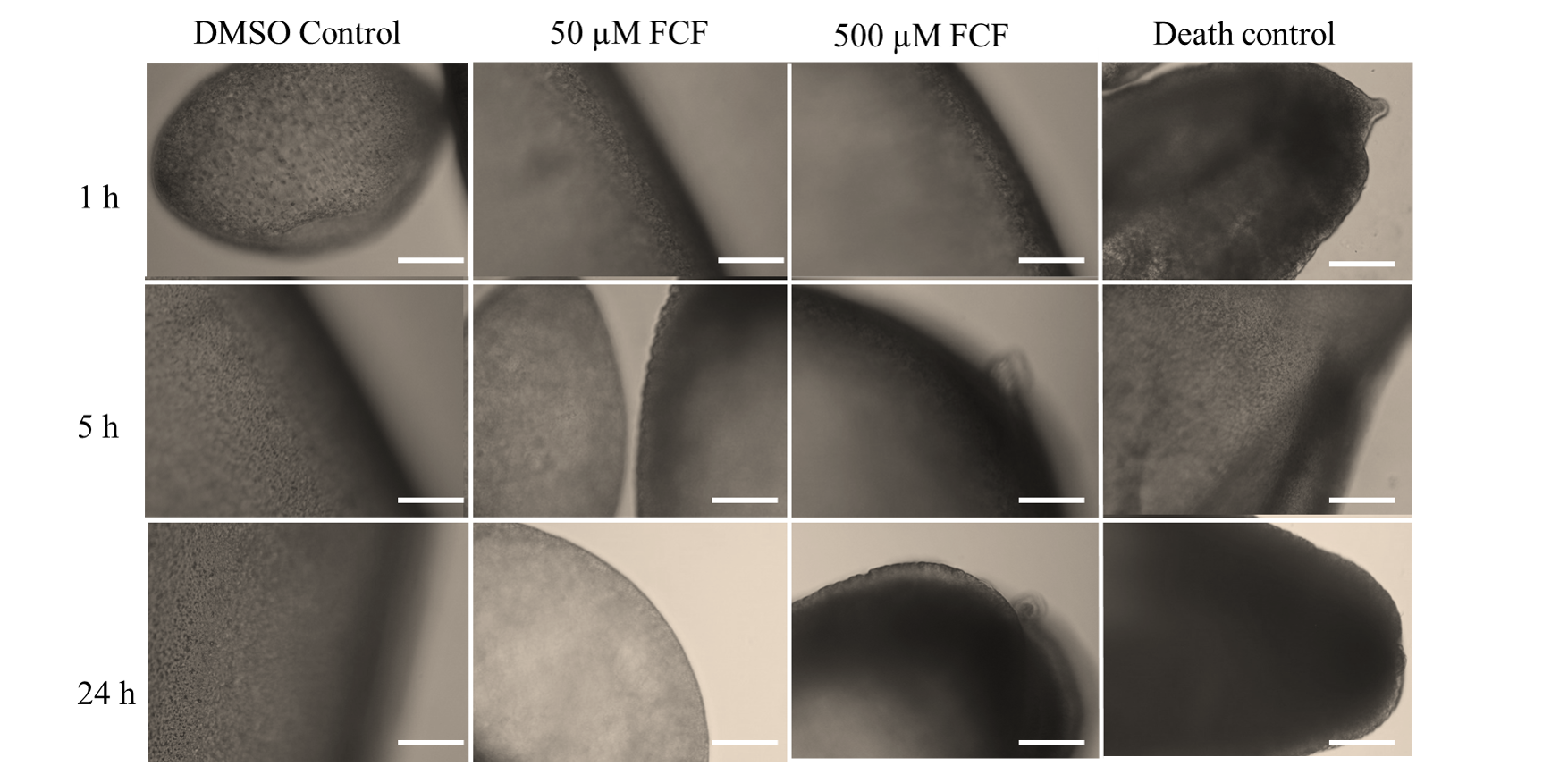

Supplement: Supplementary Materials — Supplementary Figure S1. Alignment of T. solium septins. A: SEPT4_TsM, B: SEPT7_Tsm, C: SEPT_Tsm, and D: SEPT10_Tsm were aligned with S. mansoni septins and human septins. The conserved septin motifs (G1, G3, G4, and SUE) are enclosed by black boxes. The percent similarities are shown at the bottom of the figure. Supplementary Figure S2. Motility of cysticerci treated with 5, 50, and 500 μM FCF compared to control after 1 h of treatment. Motility in cysticerci was reduced upon exposure to the highest concentrations of FCF (50 and 500 μM) in comparison with that of the control parasites (treated with DMSO), which exhibited normal motility. Supplementary Figure S3. Motility of cysticerci treated with 5, 50, and 500 μM FCF compared to control after 24 h of treatment. Upon exposure to the highest concentrations of FCF (50 and 500 μM), motility was reduced in comparison with that of the control parasites (treated with DMSO), which exhibited normal motility. Supplementary Figure S4. Reversible effect of treatment with 50 μM FCF compared to control after 5 h of treatment. Cysticerci recovered their motility slowly after changing the medium to FCF-free medium. Supplementary Figure S5. Reversible effect treatment with 50 μM FCF compared to control after 24 h of treatment. After 24 h of exposure to FCF, cysticerci did not recover their motility after changing the medium to FCF-free medium. Supplementary Figure S6. Viability of cysticerci treated with 50 and 500 μM FCF. Bright-field observations. Viability was analyzed by SYTOX green. (A) Quantification of RFUs of cysticerci treated with DMSO (control) or FCF (50 or 500 μM) compared with that of dead control cells. (B) Microscopic analysis of parasites incubated with SYTOX green after FCF exposure. Scale bars represent 500 µm. Supplementary Figure S7. Indirect effect of FCF on actin and tubulin in cysticerci of T. crassiceps. Cysticerci were treated with 500 μM FCF and then evaluated for the expression of actin using phalloidi [file 8578936.f1.zip › 8578936.f1/S6.tif]

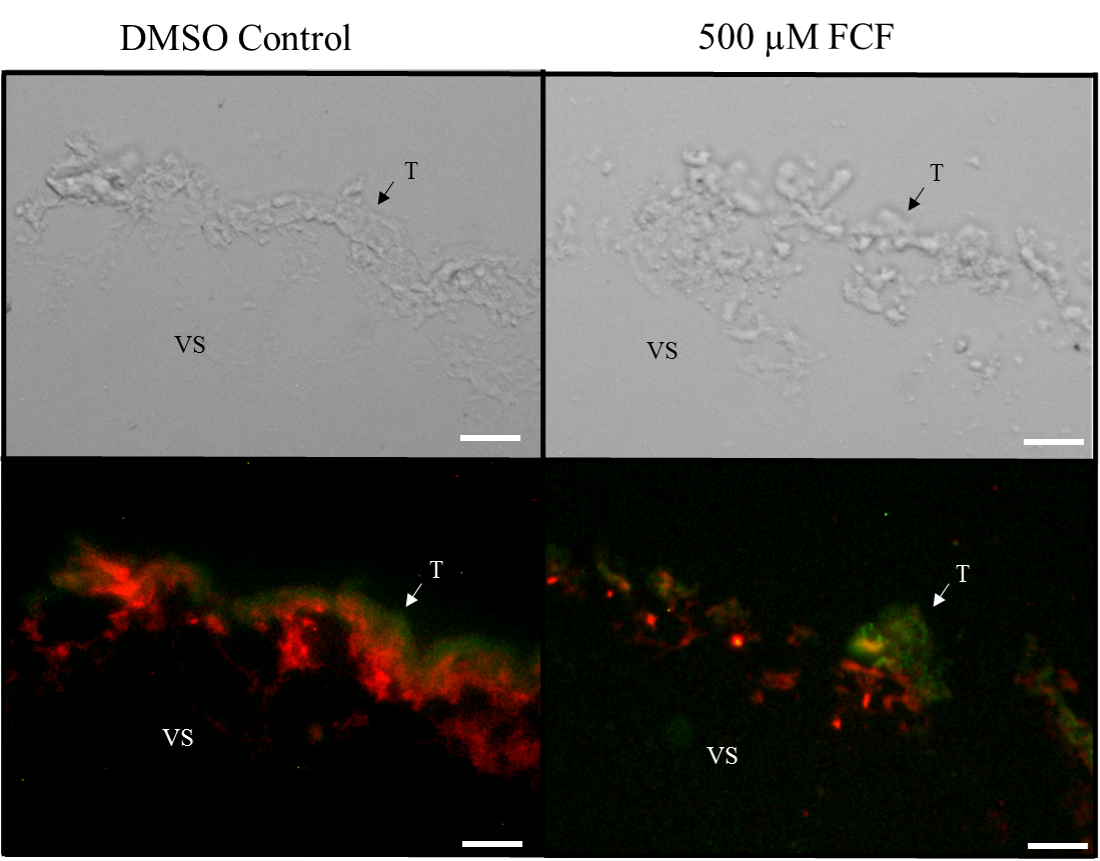

Supplement: Supplementary Materials — Supplementary Figure S1. Alignment of T. solium septins. A: SEPT4_TsM, B: SEPT7_Tsm, C: SEPT_Tsm, and D: SEPT10_Tsm were aligned with S. mansoni septins and human septins. The conserved septin motifs (G1, G3, G4, and SUE) are enclosed by black boxes. The percent similarities are shown at the bottom of the figure. Supplementary Figure S2. Motility of cysticerci treated with 5, 50, and 500 μM FCF compared to control after 1 h of treatment. Motility in cysticerci was reduced upon exposure to the highest concentrations of FCF (50 and 500 μM) in comparison with that of the control parasites (treated with DMSO), which exhibited normal motility. Supplementary Figure S3. Motility of cysticerci treated with 5, 50, and 500 μM FCF compared to control after 24 h of treatment. Upon exposure to the highest concentrations of FCF (50 and 500 μM), motility was reduced in comparison with that of the control parasites (treated with DMSO), which exhibited normal motility. Supplementary Figure S4. Reversible effect of treatment with 50 μM FCF compared to control after 5 h of treatment. Cysticerci recovered their motility slowly after changing the medium to FCF-free medium. Supplementary Figure S5. Reversible effect treatment with 50 μM FCF compared to control after 24 h of treatment. After 24 h of exposure to FCF, cysticerci did not recover their motility after changing the medium to FCF-free medium. Supplementary Figure S6. Viability of cysticerci treated with 50 and 500 μM FCF. Bright-field observations. Viability was analyzed by SYTOX green. (A) Quantification of RFUs of cysticerci treated with DMSO (control) or FCF (50 or 500 μM) compared with that of dead control cells. (B) Microscopic analysis of parasites incubated with SYTOX green after FCF exposure. Scale bars represent 500 µm. Supplementary Figure S7. Indirect effect of FCF on actin and tubulin in cysticerci of T. crassiceps. Cysticerci were treated with 500 μM FCF and then evaluated for the expression of actin using phalloidi [file 8578936.f1.zip › 8578936.f1/S7.tif]
